# Emotional competence of nurses and therapeutic communication towards the person with manifestations of mental disorder

**DOI:** 10.1192/j.eurpsy.2023.409

**Published:** 2023-07-19

**Authors:** C. Laranjeira, D. Moreira, L. Gomes

**Affiliations:** ^1^ School of Health Sciences; ^2^ciTechCare, Polytechnic of Leiria, Leiria; ^3^ Hospital Divino Espírito Santo; ^4^School of Health Sciences, Universidade dos Açores, Açores, Portugal

## Abstract

**Introduction:**

Emotional competence is fundamental in the communication that nurses establish with the people they relate to, and therapeutic communication is a crucial tool in nursing care.

**Objectives:**

This study aimed to understand the influence of nurses’ emotional competence in therapeutic communication with people with manifestations of mental disorder in a portuguese general hospital.

**Methods:**

A descriptive-correlational and transversal study was performed. The sample consisted of 171 nurses from a general hospital in the Azores. Data were collected between May and July 2021, using an instrument that includes the sociodemographic and professional questionnaire, the Veiga Scale of Emotional Competence–Reduced version [EVCE-Reduced] (Veiga-Branco, 2021) and the questionnaire “Therapeutic communication: use by nurses” (adapted from Coelho, 2015).

**Results:**

Nurses, mostly female, aged between 31 and 40 years, show moderate levels of emotional competence. Specialized nurses with more time in professional practice demonstrate to be more emotionally literate. The EVCE-Reduced demonstrates good internal reliability. Self-motivation was the most predictive capacity of emotional competence, and self-awareness the capacity with the highest average. Three communication profiles were delineated, with Profile 1 (Nurse centered on the person and on themselves) the most representative. Although there is no correlation between emotional competence and therapeutic communication, it was evident that the more empathetic nurses perceive themselves, the more they use therapeutic communication techniques; and the better they manage emotions, the more they mobilize therapeutic communication techniques and attitudes towards people with manifestations of mental disorder.

**Conclusions:**

In view of the results, it is important to deepen the way in which therapeutic communication and emotional competence are manifested in professional practice, what facilitates or inhibits its expression, and how its development can be enhanced. In this sense, health institutions must implement actions for the education and training of nurses, through the contribution of specialized practice in Mental Health Nursing, allowing for the improvement of emotional literacy and performance in work contexts.

**References:**

- Coelho, M. T. (2015). Comunicação terapêutica em enfermagem: utilização pelos enfermeiros. Porto University [doctoral thesis]. Available from: https://repositorio-aberto.up.pt/bitstream/10216/82004/2/33990.pdf (accessed on 15 september 2022); - Veiga-Branco, M. A. (2021). Competência emocional, os Dados Tomam a Palavra. Jornadas da Associação Portuguesa de Inteligência Emocional. Bragança: Instituto Politécnico de Bragança;

**Disclosure of Interest:**

None Declared

